# Nutritional status of tribal and non-tribal adults in rural Bangladesh: A comparative study

**DOI:** 10.1371/journal.pone.0287625

**Published:** 2023-07-14

**Authors:** Md. Reazul Karim, Abu Sayed Md. Al Mamun, Md. Ripter Hossain, Md. Nurul Islam, Md. Masud Rana, Md. Abdul Wadood, Kaushik Bose, Premananda Bharati, Md. Golam Hossain

**Affiliations:** 1 Health Research Group, Department of Statistics, University of Rajshahi, Rajshahi, Bangladesh; 2 Department of Anthropology, Vidyasagar University, Midnapore, West Bengal, India; 3 Biological Anthropology, Indian Statistical Institute, Kolkata, West Bengal, India; West Bengal State University, INDIA

## Abstract

**Background:**

Nutritional status is an important indicator of health status among adults. However, to date, there exists scanty information on the nutritional status of tribal populations of Bangladesh. The aim of the study was to investigate the nutritional status of tribal (T) and non-tribal (NT) adult people living in the rural area of Rajshahi district, Bangladesh.

**Methods:**

A total of 420 (72 T and 348 NT) households were studied. The samples were selected using multistage stratified sampling with proportional allocation. The nutritional status of adults was measured using body mass index (BMI). Descriptive statistics, t-test, ANOVA and Z-proportional test were utilized for data analysis.

**Results:**

The study revealed that 8.3% and 9.2% of T and NT men were suffering from under nutrition respectively, while the corresponding figures in women were 12.5% and 10.1% respectively. Overall, 11.1% and 27.0% men, and 13.9% and 29.3% women T and NT were over-nourished respectively. The rate of over nutrition among T was significantly (p<0.05) higher than NT for both sexes. The mean weight and BMI of the NT men were significantly (p<0.01) higher than T men. The mean weight, height and BMI of NT women were higher (p<0.05) than T women. ANOVA demonstrated that the variation in BMI among education levels of NT men and the variation among occupation for both ethnicities were significant (p<0.01). The variation in BMI among education levels and occupation of T and NT women were significant (p<0.05), moreover ordinal logistic regression model demonstrated that hygienic toilet facilities and father’s occupation were predictors of nutritional status. The interaction effects of education and occupation, and education and household monthly income on BMI were significant (p<0.01) for T men and both T and NT women (p<0.05).

**Conclusions:**

The prevalence of over-nutrition among NT is higher than T for both sexes. Some socio-economic and demographic factors were found as predictors of malnutrition. At least 12 of the 17 Sustainable Development Goals (SDGs) contain indicators that are highly related to nutrition, our findings can help Bangladesh Government for achieving SDGs by 2030. Appropriate nutritional intervention and awareness programmes can be initiated by the Government to ameliorate the burden of malnutrition among adults in the country.

## Introduction

Malnutrition of adults is a persistent global public health concern that causes different types of diet-related non-communicable diseases [1]. The nutritional status of adults is often neglected in low-income countries [2]. Dual malnutrition (over and under nutrition) and nutrition transition are coexistent in the low- and middle-income countries [3].

In 2016, WHO reported that globally more than 1.9 billion (39%) adults (age ≥18 years) were overweight; of them, over 650 million (13%), (11% men and 15% women) were obese. The prevalence of overweight among adults had increased from 20.2% in 1975 to 30.1% in 2016 [4].

An earlier study reported that most of the countries in the world had been experiencing an increase in the rates of overweight and obesity [5]. In Bangladesh, 30.4%, 18.9%, and 4.6% of married adults (aged≥35 years) were underweight, overweight and obese respectively [6]. Another study reported that the prevalence of underweight, normal weight and overweight was 24.1%, 46.7% and 29.2% respectively among married women in Bangladesh [7].

It is estimated that the prevalence of underweight will decrease in the world population, while overweight and obesity will increase [8]. The risk for illness, poor physical performance, lethargy, and even death are increased with the increase of malnutrition, underlying host factors are political instability, low economic development, inequality and dimensions of globalization [9].

Over the last 20 years, the double burden of malnutrition, where overweight and obesity exist with under-nutrition, has increased the incidence of diet-related non-communicable diseases [10]. Nutritional status is dependent on social, biological, environmental and cultural factors, and the context of food security. Women eat last and least throughout their whole lives, which is the age-old tradition in South Asia. On the other hand, women in better socioeconomic groups are more likely to become obese than men due to the availability of food, along with their lack of knowledge on balanced diet resulting from the low level of education, cultural discrimination and limited mobility [10].

Tribes are known for their distinct culture, belief system, economic activities, political system, dietary habits, customary laws and languages [11]. Bangladesh has several T populations and they constitute about 1% of the total population. Distinctive geographic location and rural lifestyles were influential factors for the T communities’ insufficient access to health care facilities [12]. Local beliefs and customs influence what food they would consume. The socio-economic needs, health-seeking behavior, practices affecting nutritional intake and aspirations differ from one ethnic community to another [13]. In general, T people are recognized as socially and economically vulnerable [14]. Inequality in health and nutrition exists between T and NT populations worldwide [15]. Social discrimination between ethnic minorities (T) and majority (NT) populations existed in Bangladesh [16]. Most of the T people living in Bangladesh are farmers and have no land of their own for cultivation [17].

In Bangladesh, most of the T people live in the Hill Tracts of Chittagong division. Some T communities also live in the plains, mostly in northern Bangladesh. In general, they are uneducated poor labourers and have poor access to sufficient foods¸ education and medical facilities compared to NT living in the same area [18]. Bangladesh Government and some non-government organizations now have policies targeting T communities and their household wealth quintile has been increasing during the last two decades [19]. The customs and culture of T communities are different from NT populations though they are living in the same region. Also, the food habits and behavior attitudes of tribes are different from the NT population [20]. The nutritional status of adults is related to socio-economic, demographic, cultural and behavioral factors. These factors can cause differences in nutritional status between T and NT populations living in the same geographical region. The household wealth quintiles, education level and communication system have been increasing during the last two decades in Bangladesh [21]. Although some researchers studied the nutritional status of NT populations in Bangladesh [2, 22, 23], to the best of our knowledge, information on the nutritional status of T residing in the plains of the country is lacking.

Therefore, the objective of this study was to investigate the nutritional status of T and NT adults living in the rural area of Rajshahi district, Bangladesh.

## Methods

### Area and population

The rural area of Rajshahi district was the study area, and all married adult T and NT people living in the same region were considered as the population of this study. This cross-sectional study was conducted from August 1, 2019 to October 30, 2019. Total area of the Rajshahi district is 2,425.37km^2^, and 17,40,578 people (men 8,72,467 and women 8,68,111) are living in rural area of this district [24]. Rajshahi district is located in the north-western part of Bangladesh and separated from India by a branch of the Ganges River (Padma branch). In northwest of Rajshahi lies the elevated and undulating Barind region; to the south is the high, well-drained Padma Valley; and a swampy depression drains the land near the city [25]. Rajshahi has one of the highest poverty rates in Bangladesh [26]. There are 9 Upazilas, 71 Unions and 1,727 villages in Rajshahi district, and about 67.07% adults with around 2.3% ethnic minority (T), most of who live in the rural area of this district, and most of them are Santal [24].

### Inclusion criteria

We considered the households located in the study area for at least five years. Only married adults (aged>18 years) currently living together in the selected households, having no serious disease and non-pregnant women were recruited.

### Sample size determination

The required sample size for this study was determined by using the formula, $n=\frac{z^{2}p(1-p)}{d^{2}}$ [27], where n = the number of samples, z = 1.96, p = 0.31, the probability of prevalence of under nutrition among non-pregnant married women in Bangladesh [28] and d = 0.05 (margin of error). The formula provided that 329 households were the minimum required sample size for the study. However, initially, 500 households were considered, and one adult married man and his wife (non-pregnant) were selected from each selected household.

### Sampling and data collection procedures

Multi-stage stratified random sampling with proportional allocation technique was used in this study for selecting 500 households. Out of nine Upazilas in the Rajshahi district, Godagari Upazila was selected purposively because most of the T people live in this Upazila. One union was selected randomly from nine unions of the selected Upazila. Based on the number of tribal and NT populations in the rural area of Godagari Upazila, initially 92 (18.4%) T and 408 (81.6%) NT households were selected by simple random sampling. From each selected household, we selected one adult married man and his wife. All information of T and NT married adults was collected from the Union Parishad office. Before collecting data, the objective of this study was discussed with the selected respondents. Some of them did not agree to provide their information, some others were living separately; some were widows or widowers, and some women were pregnant; all those households were excluded. Finally, 420 households were considered for this study with a non-response rate of 16% (T 21.74%; and NT 14.71%), 72 (17.10%) and 348 (82.90%) were T and NT households respectively. The total number of samples was 420 X 2 = 840, (72 X 2 = 144 T and 348 X 2 = 696 NT). In case of more than one couple (husband and wife) in the same household, we selected one couple by lottery and if a male had more than one wife, we selected one wife by lottery. Information was collected from selected samples using a self-developed questionnaire. The draft questionnaire was sent to five experts who were doing research in health sciences, and we followed experts’ opinions and suggestions to finalize the questionnaire. The mother tongue of our participants is Bangla; the questionnaire was translated into Bangla to make it easily understandable for them. The present authors carefully checked the translated version of the questionnaire. A pilot study was conducted for checking the consistency and validity of questionnaire by Cronbach’s alpha, and the researchers’ did not find any problem.

### Outcome variable

Nutritional status was the dependent variable in this study, and it was measured by BMI. BMI was calculated using the following formula: BMI = weight (kg)/ {height (m)}^2^. Digital scales and a portable stadiometer were used to measure their weight and height respectively. Measurements of individuals were taken without shoes and wearing light clothes. Height and weight were measured to the nearest 1 cm and 0.1 kg respectively. According to WHO, the nutritional status of adults was classified as: (i) under-nutrition (BMI<18.5 kg/m^2^), (ii) normal weight or healthy (18.5≤BMI<25 kg/m^2^) and (iii) over nutrition (BMI≥25 kg/m^2^) [29].

### Independent variables

The following socio-economic and demographic variables were considered as independent variables: age, gender, education level, occupation, religion, household monthly income (salary allowance, agriculture, remittance, business, grants, other sources), toilet facilities (hygienic toilet (toilet with septic tank / offset pit toilet / ring-lab direct pit (with water seal), and unhygienic toilet (ring slab direct pit (without water seal) / hanging / hole toilet), in open space (open space/ bush / river / canal / pond bank)), having electricity, having television, having safe water access (Yes, sources of drinking water: tube well, protected dug well, piped household connection (tested arsenic and bacteria), No, drinking water sources: unprotected dug well, surface (river, dam, lake, pond, stream, canal, irrigation channel etc.). The classification of the variables is described in Table 1. Most of the variables were selected on the basis of the previous study in Bangladesh [23].

**Table 1:** Socio-economic and demographic characteristic of tribal and non-tribal.

Variables	Groups	Ethnic Group	
		Tribal N (%)	Non-tribal N (%)
**Age group (Men)**	≤ 30 years	3 (4.4)	30 (8.8)
	31–35 years	13 (19.1)	93 (27.4)
	36–40 years	13 (19.1)	97 (28.5)
	> 40 years	39 (57.4)	120 (35.3)
**Age group (women)**	≤ 30 years	13 (18.1)	77 (22.2)
	31–35 years	29 (40.3)	158 (45.5)
	36–40 years	13 (18.1)	72 (20.7)
	> 40 years	17 (23.6)	40 (11.5)
**Education level (Men)**	Uneducated	11 (15.3)	53 (15.2)
	Primary	36 (50.0)	156 (44.8)
	Secondary	19 (26.4)	95 (27.3)
	Higher education	6 (8.3)	44 (12.6)
**Education level (women)**	Uneducated	5 (6.9)	19 (5.5)
	Primary	29 (40.3)	122 (35.1)
	Secondary	34 (47.2)	185 (53.2)
	Higher education	4 (5.6)	22 (6.3)
**Occupation (Men)**	Labour (Agriculture)	39 (54.2)	143 (41.1)
	Labour (Non-agriculture)	17 (23.6)	85 (24.4)
	Farmer	5 (6.9)	40 (11.5)
	Business	4 (5.6)	40 (11.5)
	Service	1 (1.4)	27 (7.8)
	Others (remittance)	6 (8.3)	13 (3.7)
**Occupation (women)**	Labour (Agriculture)	53 (73.6)	5 (1.4)
	Service	3 (4.2)	5 (1.4)
	Housewife	16 (22.2)	338 (97.2)
**Religion**	Muslim	0 (0)	348 (100)
	Non-Muslim	72 (100)	0 (0)
**Having hygienic toilet**	Yes	42 (58.3)	200 (57.5)
	No	30 (41.7)	148 (42.5)
**Having safe water access**	Yes	69 (95.8)	321 (92.2)
	No	3 (4.2)	27 (7.8)
**Having electricity**	Yes	58 (80.6)	273 (78.4)
	No	14 (19.4)	75 (21.6)
**Having Television**	Yes	72 (100.0)	343 (98.6)
	No	0 (0.00)	5 (1.4)
**Household monthly income (BDT)**	<7,500	7 (9.7)	48 (13.8)
	7,501–10,000	29 (40.3)	114 (32.8)
	10,001–15,000	31 (43.1)	118 (33.9)
	>15,000	5 (6.9)	68 (19.5)

### Ethics approval and consent to participate

Ethical approval for this study was obtained from the Ethical Review Committee of Institute of Biological Sciences, University of Rajshahi, Rajshahi-6205, Bangladesh (Memo No. 69/320/IAMEBBC/IBSC). Before collecting data, we discussed about the objectives of the study with selected participants, written consent was obtained from them, and their right to remain or opt out if they feel uncomfortable.

### Statistical analysis

Descriptive statistics was carried out to find the prevalence of malnutrition among T and NT adults and t-test was used to find the differences in height, weight and BMI between two groups (T and NT). Moreover, two-way analysis of variance (ANOVA) was used to find the differences in BMI in case of more than two groups, and Z-proportional test was also used to find the differences between two proportions. The normality and homogeneity of group variances were checked by using Kolmogorov–Smirnov nonparametric test and Levene test respectively for the validity of our data in parametric (t-test and ANOVA) test [30]. Finally, ordinal logistic regression model was utilized to find the effect of socio-economic and demographic factors on nutritional status among tribal and non-tribal adults. Simple ordinal logistic model was used to select the independent variable (p-value<0.20) for multiple model, A statistical package, SPSS (Version IBM, 22) was used to carry out the entire analysis. A value of p<0.05 was considered statistically significant in the analysis.

## Results

A total number of 420 households and one couple (husband and non-pregnant wife) from each household were selected for this study. Out of 420 households, 72 (17.10%) and 348 (82.90%) households were T and NT respectively. In this study, 144 (72 adult married men and 72 women) of T and 696 (348 men and 348 women) of NT participated. The mean age of men and women were 38.90±6.57 years with range of 23 to 59 years, and 33.91±5.80 years with range of 20 to 54 years respectively.

Table 1 revealed that the highest number (57.4%) of the T men were above 40 years, while the highest number (35.3%) of NT were above 40 years. Similarly, the highest number of T (40.3%) and NT (45.4%) women was in the age group of 31–40 years. More than 54% and 41% of T and NT men were working in agriculture respectively, while only 5.6% of T and 11.5% of NT were doing business respectively. Overall, 50.0% and 44.8% of T and NT men had primary education, 15.3% and 15.2% of T and NT were uneducated, and 5.6% of T and 12.6% of NT men were higher educated. On the other hand, 40.3% of T women completed primary education and 47.2% were secondary educated while 35.1% of NT women were primary and 53.2% were secondary educated. More than 73% of T women were involved as labor in agriculture while the corresponding figure was only 1.4% among NT women. On the other hand, more than 97% of NT women were housewives while only 22.2% of T women were housewives. It was observed that 100% of households of T community in Rajshahi district were non-Muslim while 100% NT were Muslims. Overall, 80.6% of T and 78.4% of NT households had electricity. It was found that more than 95% of tribal households used safe water for drinking while 92.2% of NT people had access to safe water. It was noted that 41.7% and 42.5% of T and NT households did not have hygienic toilets. Almost all households of both T and NT had televisions. Monthly income of more than 40% and 43% of T families were 7,501–10,000 Taka and 10,001–15000 Taka respectively, while 32.8% and 33.9% of NT families had monthly income of 7,501–10,000 Taka and 10,001–15000 Taka respectively (Table 1).

It was observed that only 8.3% and 9.2% of T and NT men were undernourished respectively, while two times higher numbers (27%) of NT men were over nourished than that of T people (11.1%). Chi-square test demonstrated that the association between ethnic groups and their nutritional status of men was significant (p<0.05). We observed that the prevalence of undernourished T women (12.5%) was more than that of NT women (10.1%), while the prevalence of over nourished among NT women (29.3%) was higher than that of T women (13.9%). The association between these two factors was significant (p<0.05). The prevalence of malnutrition (under nutrition and over nutrition) among women was higher than men for both groups (Table 2).

**Table 2:** The association between nutritional status and ethnic groups by gender.

Gender	Ethnic Groups	Under nourished, N (%)	Healthy, N (%)	Over nourished, N (%)	*χ*^2^-value	p-value
**Men**	Tribal	6(8.3)	58(80.6)	8(11.1)	8.774	0.012
	Non-tribal	32(9.2)	222(63.8)	94(27.0)		
**Women**	Tribal	9(12.5)	53(73.6)	10(13.9)	7.259	0.027
	Non-tribal	35(10.1)	211(60.6)	102(29.3)		

Z-proportional test showed that the under and over nutrition of NT men were significantly (p<0.05) higher than that of T men. For women, over nutrition of NT was significantly (p<0.05) higher than T women. It was noted that the proportions of normal weight among T adults were significantly (p<0.05) higher than NT adults for both sexes (Table 3).

**Table 3:** Comparison of nutritional status between tribal and non-tribal adults.

Gender	Nutritional status	Tribal, N (%)	Non-tribal, N (%)	z- value	p-value
Men	Under nutrition	6 (8.3)	32 (9.2)	0.10	0.9438
	Normal	58 (80.6)	222 (63.8)	2.40	0.0152
	Over nutrition	8 (11.1)	94 (27.0)	1.80	0.0450
Women	Under nutrition	9 (12.5)	35 (10.1)	0.20	0.8347
	Normal	53 (73.6)	211 (60.6)	1.90	0.0250
	Over nutrition	10 (13.9)	102 (29.3)	1.70	0.0490

Kolmogorov–Smirnov non-parametric test showed that our dependent variables weight, height and BMI were normally distributed; data satisfied the standard assumption of the t-test. It was found that the mean weight of NT men (61.76±8.18 kg) was significantly (p<0.01) higher than that of T (58.12±6.51 kg). The difference of mean height between T and NT men was not significant (p>0.05). The mean BMI of NT men (22.91±3.18 kg/m^2^) was significantly (p<0.01) higher than that of T men (21.63±2.02 kg/m^2^). For women, the mean weight (54.54 ± 9.51 kg) (p<0.01), height (153.73±5.05 cm) (p<0.05) and BMI (23.06 ±3.74 kg/m^2^) (p<0.01) of NT women were significantly higher than those of T women’s weight (50.717.75 kg), height (152.294.81 cm, and BMI (21.822.87 kg/m^2^) respectively (Table 4).

**Table 4:** Comparison in nutritional status between tribal and non-tribal married adult men and women.

Gender	Measurements	Tribal	Non-tribal	p-value
Men	Weight (kg)	58.12±6.507	61.76±8.18	0.001
	Height (cm)	163.90±6.19	164.39±6.24	0.554
	BMI(kg/m^2^)	21.63±2.02	22.91±3.18	0.002
Women	Weight (kg)	50.71±7.75	54.54±9.51	0.001
	Height (cm)	152.29±4.81	153.73±5.05	0.026
	BMI(kg/m^2^)	21.82±2.87	23.06±3.74	0.008

Before using ANOVA, we checked the standard assumptions (normality and homogeneity of group variances) underlying the ANOVA model. We already checked the normality of BMI, it was necessary to check the homogeneity of group variances for ANOVA model. The Levene test demonstrated that the data were homogeneous. Thus, the data satisfied the standard assumptions of the ANOVA model. ANOVA showed that the variation of mean BMI of NT men was significant (p<0.05) among their educational levels, but no significant difference of mean BMI was observed in T adults men (p>0.05). Mean BMI was significantly (p<0.05) varied by occupation for both T and NT men. For T women, there was a significant (p<0.05) variation of mean BMI between their educational levels, while no significant (p>0.05) difference in mean BMI was observed among NT women between education levels. The mean BMI was significantly (p<0.05) varied by occupation of the NT women but no significant difference was observed in tribal due to their occupation. The variation of mean BMI among household income groups of T women was significant (p<0.05). It was found that the mean BMI increased with increasing household income of NT women and T men while there was no clear pattern in BMI of NT men; the variations of BMI were not statistically significant (p>0.05). Two way ANOVA demonstrated that the interaction effects of education and occupation, and education and household monthly income on BMI were significant (p<0.01). However the effects of occupation and household monthly income was not significant (p>0.05) for T men while the effects of these interactions were not significant for NT men (p>0.05). For women, the interaction effects of education and occupation, and education and household monthly income on BMI were significant (p<0.05) for T and NT, respectively. Other interactions were not significant (p>0.05) (Table 5).

**Table 5:** Comparison of BMI by socio-economic factor.

Gender	Variables	Groups	Tribal		Non-tribal	
			Mean ±SD (BMI)	F-value	Mean ±SD (BMI)	F-value
Men	Education	Uneducated	20.91±2.19	1.24	23.39±3.29	6.74**
		Primary	21.52±2.08		22.32±2.97	
		Secondary	21.71±2.01		22.84±3.11	
		Higher education	22.95±1.05		24.62±3.36	
	Occupation	Labour (Agriculture)	21.15±1.61	5.19**	22.51±3.26	3.06**
		Labour (Non-agriculture)	21.80±1.28		22.96±2.86	
		Farmer	20.87±4.06		22.14±2.58	
		Business	25.47±0.86		23.90±3.63	
		Service	23.20±2.13		24.10±3.37	
		Others	23.71±3.59		25.19±1.81	
	Household monthly income (in BDT)	<7,500	20.77±0.62	1.61	23.03±2.99	0.70
		7,501–10,000	21.22±1.97		22.64±3.15	
		10,001–15,000	21.87±2.17		22.86±3.46	
		>15,000	23.06±1.02		23.33±2.84	
	**Interaction effect**					
	Education * occupation			7.58**		0.76
	Education * household monthly income (in BDT)			3.89**		1.67
	Occupation * household monthly income (in BDT)			0.86		0.68
Women	Education	Uneducated	20.03±1.24	3.32*	21.78±2.37	1.85
		Primary	20.89±2.74		22.78±3.90	
		Secondary	22.79±2.94		23.23±3.75	
		Higher education	22.49±1.73		24.28±3.30	
	Occupation	Labour (Agriculture)	21.44±2.92	2.00	19.55±3.80	2.72*
		Service	21.66±0.60		24.70±2.98	
		Housewife	23.07±2.69		23.09±3.72	
	Household monthly income (in BDT)	<7,500	23.23±4.20	2.23*	22.39±3.93	0.60
		7,501–10,000	22.37±2.86		22.86±3.76	
		10,001–15,000	20.86±2.41		23.00±3.79	
		>15,000	22.52±2.44		23.59±3.49	
	**Interaction effect**					
	Education * occupation			3.65*		0.40
	Education * household monthly income (in BDT)			1.81		2.33*
	Occupation * household monthly income (in BDT)			2.08		1.32

N.B.

**: 1% and

*: 5% level of significance.

It was found that the mean of BMI for all categories of education levels of NT men were higher than T, however only the difference between uneducated groups was significant (p<0.05). The mean BMI of NT labour (agriculture) was significantly (p<0.05) higher than tribal labour (agriculture). Poor (family income, 7,501–10,000 Taka) NT men had significantly (p<0.05) higher mean BMI than T. Primary educated NT women had significantly (p<0.05) higher BMI than that of T women, also NT women living in upper middle class family (family monthly income, 10,001–15,000 taka) had significantly (p<0.01) higher BMI than that T women (Table 6).

**Table 6:** Comparison in BMI between tribal and non-tribal by different categories of socio-economic factors.

Gender	Variables	Groups	BMI of Tribal	BMI of Non-tribal	p-value
			Mean ±SD	Mean ±SD	
Men	Education	Uneducated	20.91±2.19	23.39±3.29	0.020
		Primary	21.52±2.08	22.32±2.97	0.127
		Secondary	21.71±2.01	22.84±3.11	0.131
		Higher Education	22.95±1.05	24.62±3.36	0.236
	Occupation	Labour (Agriculture)	21.15±1.61	22.51±3.26	0.012
		Labour (Non-agriculture)	21.80±1.28	22.96±2.86	0.105
		Farmer	20.87±4.06	22.14±2.58	0.336
		Business	25.47±0.86	23.90±3.63	0.397
		Service	23.20±2.13	24.10±3.37	0.715
		Others	23.71±3.59	25.19±1.81	0.241
	Household monthly income (in BDT)	<7,500	20.77±0.62	23.03±2.99	0.053
		7,501–10,000	21.22±1.97	22.64±3.15	0.022
		10,001–15,000	21.87±2.17	22.86±3.46	0.132
		>15,000	23.06±1.02	23.33±2.84	0.834
Women	Education	Uneducated	20.03±1.24	21.78±2.37	0.129
		Primary	20.89±2.74	22.78±3.90	0.014
		Secondary	22.79±2.94	23.23±3.75	0.630
		Higher Education	22.49±1.73	24.28±3.30	0.201
	Occupation	Labour (Agriculture)	21.44±2.92	19.55±3.80	0.182
		Service	21.66±0.60	24.70±2.98	0.141
		Housewife	23.07±2.69	23.09±3.72	0.983
	Household monthly income (in BDT)	<7,500	23.23±4.20	22.39±3.93	0.602
		7,501–10,000	22.37±2.86	22.86±3.76	0.513
		10,001–15,000	20.86±2.41	23.00±3.79	0.003
		>15,000	22.52±2.44	23.59±3.49	0.504

Simple and multiple ordinal logistic regression models were used to find the effect of socio-economic and demographic factors on nutritional status of tribal and non-tribal adult population. The univariate model provided that having hygienic toilet (p<0.20), having electricity (p<0.20) and fathers’ occupation (p<0.20 would be considered as independent variables in multiple model for both tribal and non-tribal; and having safe water access (p<0.20) was also considered as independent variables in multiple model for tribal. After controlling the effect of other variables, multiple ordinal logistic model demonstrated that household having hygienic toilet was more likely to get over nutrition for tribal [AOR = 0.207, 95% CI: 0.062–0.689, p<0.05] and for non-tribal [AOR = 0.630, 95% CI:0.386–0.986, p<0.05]. Fathers doing job or business (others) were more likely to have over nourished compared to farmers for tribal [AOR = 0.005, 95% CI: 0.001–0.071, p<0.01] and non-tribal [AOR = 0.327, 95% CI: 0.194–0.551, p<0.01] (Table 7).

**Table 7:** Ordinal logistic regression analysis of factors influencing nutritional status of tribal and non-tribal adults.

		Ethnic Group				Ethnic Group			
		Tribal		Non-tribal		Tribal		Non-tribal	
Variable	Group	OR, 95% CI of OR (Lower-Upper)	p-value	OR, 95% CI of OR (Lower-Upper)	p-value	AOR, 95% CI of AOR(Lower-Upper)	p-value	AOR, 95% CI of AOR(Lower-Upper)	p-value
Age group (Ordinal)		0.989(0.704–1.390)	0.989	1.104(0.793–1.538)	0.558				
Education level (Ordinal)		0.882(0.524–1.483)	0.636	1.051(0.796–1.387)	0.728				
Household monthly income (BDT) (Ordinal)		0.457(0.204–1.024)	0.057	1.077(0.798–1.455)	0.628				
Having hygienic toilet (Nominal)	No Vs Yes^R^	0.501(0.284–0.883)	0.017	0.654(0.566–0.755)	0.001	0.207(0.062–0.689)	0.010	0.630(0.386–0.986)	0.045
Having safe water access(Nominal)	No Vs Yes^R^	0.189(0.90–0.400)	0.001	0.968(0.450–2.083)	0.934	0.111(0.008–1.457)	0.094		
Having electricity (Nominal)	Yes Vs No^R^	2.129(1.439–3.151)	0.001	1.241(1.128–1.364)	0.001	2.474(0.636–9.620)	0.191	1.293(0.731–2.287)	0.377
Gender (Nominal)	Male Vs Female^R^	1.067(0.489–2.325)	0.871	0.942(0.748–1.186)	0.613				
Fathers’ occupation (Nominal)	Farmer Vs Others^R^	0.009(0.001–0.096)	0.001	0.315(0.189–0.525)	0.001	0.005(0.001–0.071)	0.001	0.327(0.194–0.551)	0.001
Mothers’ occupation (Nominal)	Housewife Vs Others^R^	1.855(0.535–6.424)	0.330	1.965(0.532–7.253)	0.311				

N.B.: OR: Unadjusted odds ratio; AOR: Adjusted odds ratio; R: Reference case; CI: Confidence interval.

## Discussion

We investigated the nutritional status among the 420 couples (T72 and NT348) who lived in the rural area of the Rajshahi district in Bangladesh. We also determined the factors which affected the nutritional status. The mean age of men and women were 38.90±6.57 years with range of 23 to 59 years, and 33.91±5.80 years with range of 20 to 54 years respectively.

### Nutritional status of non-tribal married adults

We found the mean BMI of NT men was 22.91±3.18 kg/m^2^ living in the rural area of Rajshahi district, Bangladesh. BDHS (2017–18) selected male sample aged ≥18 years from overall Bangladesh and measured their height and weight, calculated BMI, they reported that the mean BMI of adult male living in rural area of Bangladesh was 21.3 kg/m^2^ (urban and rural together was 21.6 kg/m^2^) which was similar to our finding [21]. We observed that the mean BMI was 23.06±3.74 kg/m^2^ among NT women. According to BDHS-2017-2018 survey, the mean BMI among rural women of Bangladesh was 22.9 kg/m^2^ and this result is similar to our study [21]. It was found that a few numbers (9.2%) of NT men was suffering from under nutrition while a large number (27.0%) were over nourished. The prevalence of under and over nutrition among Bangladeshi married men living in rural environment were 22.0% and 14.9% as was reported by BDHS (2017–2018). Both these rates were significantly different from our findings. This could be due to different geographical location, customs and cultures. We observed that 29.3% of women were suffering from over nutrition while 10.1% were under nourished. A nationally representative survey (BDHS 2017–2018) reported that 28.1% women living in rural environment were over nourished [21], which was similar to our finding. BDHS 2017–2018 also reported that 13.2% of rural women were suffering from under nutrition [21], which was also similar to our result. The proportion of under nourished among married women in Bangladesh has been decreasing but the rate of over nourished has been rapidly increasing due to increasing household wealth quintile and women education level during the last two decades [21]. In India, the existence of dual burden of malnutrition in the adult population was found in six states, showing a declining trend in underweight and increasing trend in overweight/obesity; overweight and obesity was linked with urbanization, life style changes and economic development [31]. Similar results had been reported from other developed and developing countries [32, 33].

### Nutritional status of tribal married adults

The mean BMI of T men was 21.63±2.02 kg/m^2^. Most of the tribal who are living in the rural areas of Rajshahi district, Bangladesh are Santal. A cross-sectional study among adult Santal people, a tribal population of Birbhum District, West Bengal, India found the mean BMI was 20.46 kg/m^2^ for men [34], which was similar to our finding. However, an earlier Indian study reported that the mean BMI of adult men tribal living in Orissa state, India was 18.4 kg/m^2^ [35]. The mean BMI of T women was 21.82±2.87 kg/m^2.^ The mean BMI of Santal women living in Birbhum District, West Bengal, India was 19.48 kg/m^2^ [34], which was less than our finding.

### Men Vs women

Our study found that the mean BMI of women (23.06 kg/m^2^) was significantly higher than that of men (22.91 kg/m^2^). These results are similar to the BDHS (2017–18) data; the mean BMI among women (23.3 kg/m^2^) was more than that in men (21.6 kg/m^2^) population in Bangladesh respectively [21]. Similar results were found in Botswana, the mean BMI was higher among women (24.46 kg/m^2^) than that in men (21.76 kg/m^2^) [36].

The mean BMI of men was 21.63±2.02 kg/m^2^ and of women were 21.82±2.87 kg/m^2^ among the tribal population in Rajshahi district of Bangladesh. Our study found that the mean BMI among NT men was slightly higher than that among T women. Similar results were found in West Bengal (Men-20.46 kg/m^2^, and Women- 19.48 kg/m^2^) and Orissa (Men-18.4 kg/m^2^; Women-17.9 kg/m^2^), India among the tribal populations [34, 35].

The proportion of over nourished women was higher than that of men for both ethnicities; the same result was found for the non-tribal population in Bangladesh [21]. Similar result was also found in another study of Bangladesh; authors reported that the prevalence of over nutrition among married women was higher than that of men [37]. It was observed that females who were nutritionally deprived as children were more prone to be obese than males and those women were more likely to be obese who had higher status but the same was not true for males in South Africa. They reported that these two factors were the most important in causing the difference in obesity rates between adult men and women [38].

### Differences in nutritional status between tribal and non-tribal

We observed that mean weight of non-tribal men was higher than that of T men but mean height was almost the same, and consequently, the mean BMI of married NT adult men was higher than that of T men. As a result, the prevalence of over nutrition among NT men was significantly higher than that of T men. Moreover, mean height, weight and BMI among NT women were higher than that of T women, consequently, the proportion of over nourished among NT women was higher than that of T women. It has been reported earlier that education level and the household wealth quintile were the most important predictors of the nutritional status of adults [23, 39]. We found that the association between ethnic groups and nutritional status among adults for both men and women was significant but the difference in BMI between T and NT by most of the socioeconomic factors were not significantly different, this could happen due to small number of samples in the under and over nutrition groups. In our study, we observed that the rate of secondary and higher education among NT men and women were higher than that among T men and women, and the household income of NT was higher than that of T people, which were the most important reasons for the difference in nutritional status between NT and T adults. Similar findings were found in the tribes of Maharashtra state of India, they are thin and lean with medium to short stature due to chronic energy deficiency and a high proportion of individuals with chronic energy deficiency corroborates with their low social and economic status [40]. Overweight and obesity was associated with the educational level of the T and NT men and women. Higher educated men and women have greater BMI among T as well as NT communities. Higher educated persons undertook business or were service holders (fixed income) and they earned high income than other occupations (unskilled labour and farmers). In the present study, we found that fathers’ occupation and household hygienic toilet facility were important predictors of nutritional status of both T and NT. It was also found that under or over nutrition status was related to the income groups; economically better-off were more likely to be overweight or obese and residents of rural areas and those from lower-income groups were more likely to be underweight, which were much close to our findings [41]. In the last decade, a considerable economic growth and social development have occurred in Bangladesh. This socioeconomic change has affected health and education of the households in Bangladesh [42].

The dual burden of malnutrition in our study area was found among both sexes of the two ethnic groups. Recently, many developing countries have entered a nutrition transition in which rapid changes in food availability and physical activity patterns have led to an upward shift in BMI. This transition has also been observed in countries in Latin America, Asia and Africa. The problem of underweight is also accompanied by increasing proportions of overweight and obese populations in Botswana [43]. Bangladesh has gained self-sufficiency in the food availability and is also experiencing numerous changes in the society, including life style and food habits, and socio-economy [44]. The under nutrition of Bangladeshi adults has been decreasing while over nutrition has been increasing during the last two decades due to increasing household wealth quintile in Bangladesh [21, 37].

### Strength and limitations of this study

The major strength of the present study is that it is the first comparative investigation which dealt with the problem of malnutrition among T and NT populations of Rajshahi district. This is the uniqueness of our study. Besides, our findings have immense utility in reducing the prevalence of malnutrition. However, the major limitation of our study is its cross-sectional nature. Moreover, it was confined to a specific geographical area and only a few socio-economic and demographic factors were considered.

## Conclusions

The level of higher and secondary education as well as household income among NT was higher than T people living in the same area. We found that prevalence of over nutrition among NT was higher than T, for both sexes. Education, occupation and household monthly income were important confounding factors of malnutrition.

### Recommendations and policy implications

Based on our findings, the Government of Bangladesh should attempt to ameliorate the problem of adult malnutrition. Special focus should be given to the modifiable factors associated with malnutrition. Such efforts can be supplemented by non-government health organizations. The role of household food security, family income and other associated factors are of paramount importance in addressing the problem of malnutrition.

## Supporting information

S1 File (DOC)

S1 Data (SAV)
