# Supplementary material for: Nutritional status of tribal and non-tribal adults in rural Bangladesh: A comparative study
Source: PLoS One. 2023 Jul 14;18(7):e0287625. doi: 10.1371/journal.pone.0287625 (PMC10348562; doi:10.1371/journal.pone.0287625)
Supplement: S1 File — (DOC) [file pone.0287625.s001.doc]

**Questionnaire**

**(Note: The information you provide will be kept completely confidential and will only be used for research purposes)**

**Survey on nutritional status among adults in Rajshahi district, Bangladesh: A household study**

**Data Entry Serial No:**

Respondent Name:

Address: Upazila: Union Ward: Village:

| Family mobile no |  |
| --- | --- |
| Date of interview |  |

**General information**

| Husband name |  | Age | Occupation | Education |
| --- | --- | --- | --- | --- |
|  |  |  |
| Wife name |  | Age | Occupation | Education |
|  |  |  |

**Anthropometrics status of family members**

| Sl |  | Date of birth | Age | Sex | Weight (kg) | Height (cm) | MUAC |
| --- | --- | --- | --- | --- | --- | --- | --- |
| 1 | Husband |  |  |  |  |  |  |
| 2 | Wife |  |  |  |  |  |  |
| 3 | Children 1 |  |  |  |  |  |  |
| 4 | Children 2 |  |  |  |  |  |  |
| 5 | Children 3 |  |  |  |  |  |  |
| 6 | Children 4 |  |  |  |  |  |  |
|  |  |  |  |  |  |  |  |
|  |  |  |  |  |  |  |  |

Age: (Full year, EPI and birth registration card view, NID)

**Household members**

| **0- 5 year** | | **6-14 year** | | **15-49 year** | | **50-64 year** | | **65+ year** | | **Total** | |
| --- | --- | --- | --- | --- | --- | --- | --- | --- | --- | --- | --- |
| Boy | Girl | Boy | Girl | Male | Female | Male | Female | Male | Female | Male | Female |
|  |  |  |  |  |  |  |  |  |  |  |  |

**Socio-economic information of the household**

| 1 | Ethnic group 1 = Tribal, 2 = Non-tribal |  |
| --- | --- | --- |
| 2 | Religion: 1 = Muslim, 2 = Hindu, 3 = Christian, 4 Buddhist, 5 = Other |  |
| 3 | Amount (in decimal) of own land in the family? Home: Agriculture: |  |
| 4 | Having electricity in house 1= yes, 2 = no |  |
| 5 | Having television in house 1 = yes, 2 = no |  |

Information about health habits

| 1 | Which is usually the main source of household drinking water? 1 = tube well, 2 = well, 3 = pipeline, 4 = pond, 5 = river, 6 = canal, 6 = bill, 6 = other (specific) |  |
| --- | --- | --- |
| 2 | If answer is a tube well/ well/ pipeline, Is it safe (good platform and drainage system, arsenic and bacteria free)? 1 = yes, 2 = no |  |
| 3 | What type of toilet do household members usually use? 1 = Hygienic toilet (toilet with septic tank / offset pit toilet / ring-lab direct pit (with water seal), 2 = non-hygienic toilet (ring slab lab direct pit (without water seal) / hanging / hole toilet), 3 = In open space (open space / bush / river / canal / pond bank) |  |
| 4 | Do all family members wash their hands with soap at all critical times (before preparing food, before eating, before feeding the baby, after going to the toilet, after the baby defecates, after picking up trash)? 1 = all the time, 2 = occasionally, 3 = never |  |
| 5 | Is there water and soap in the handwashing area (from the observation of the data collector)? 1 = yes, 2 = no |  |

**Household income and expenditure figures (monthly)**

| Sl | **Income** | **BDT** | Sl | **Expenditure** | **BDT** |
| --- | --- | --- | --- | --- | --- |
| 1 | Salary |  | 1 | House rent |  |
| 2 | Agriculture |  | 2 | Rice |  |
| 3 | Vegetables |  | 3 | Education |  |
| 4 | Cattle |  | 4 | Medicine |  |
| 5 | Chicken |  | 5 | Transport |  |
| 6 | Hand work |  | 6 | Mobile bill |  |
| 7 | Remittance |  | 7 | Fuel |  |
| 8 | Business |  | 8 | Furniture |  |
| 9 | Grant |  | 9 | Festival |  |
| 10 | Loan |  | 10 | Loan recovery |  |
| 11 | Other |  | 11 | Saving |  |
|  |  |  | 12 | Tax |  |
|  |  |  | 13 | Gift |  |
|  |  |  | 14 | Other |  |
|  | Total BDT |  |  | Total BDT |  |

**Household food security**

| 1 | Has no one in the family taken any food in the last 30 days due to lack of money to buy food? 1 = yes, 2 = no |  |
| --- | --- | --- |
| 2 | How many times has this happened in the last 30 days? 1 = rarely (1-2 times), 2 = sometimes (3-10 times), 3 = often (more than 10 times) |  |
| 3 | Have you or anyone in your family gone to bed hungry in the last 30 days without any food? 1 = yes, 2 = no |  |
| 4 | How many times has this happened in the last 30 days? 1 = rarely (1-2 times), 2 = sometimes (3-10 times), 3 = often (more than 10 times) |  |
| 5 | Have you or anyone in your family gone to bed hungry day and night without taking any food in the last 30 days due to lack of food? 1 = yes, 2 = no |  |
| 6 | How many times has this happened in the last 30 days? 1 = rarely (1-2 times), 2 = sometimes (3-10 times), 3 = often (more than 10 times) |  |

**Household food items (what kind of food you ate in the last 24 hours)**

| **#** | **Food ingredients**  **(Tick where applicable)** | Breakfast | Snack | Lunch | Snack | Dinner | Snack |
| --- | --- | --- | --- | --- | --- | --- | --- |
| 1 | Starch / staple food (rice, wheat, bread, porridge, corn) |  |  |  |  |  |  |
| 2 | Roots and tubers (potatoes, sweet potatoes, potato pulses) |  |  |  |  |  |  |
| 3 | Vegetables (Carrots, okra, gourds, pumpkins, mushrooms, radishes, tomatoes, barley / flower copy, beans) |  |  |  |  |  |  |
| 4 | Fruits (banana, guava, mango, pineapple, blackberry, watermelon, kamaranga, apple, quince fruit, jackfruit) |  |  |  |  |  |  |
| 5 | Meat (beef, goat, chicken) |  |  |  |  |  |  |
| 6 | Eggs (chicken, hen, quail) |  |  |  |  |  |  |
| 7 | Fish and seafood (any type of seafood, dried fish, crab) |  |  |  |  |  |  |
| 8 | Pulses / Almonds (e.g. Almonds, Soybeans, Lentils, Mugs, Masakalai) |  |  |  |  |  |  |
| 9 | Milk and dairy foods (any type of milk and dairy foods such as yogurt, ice cream) |  |  |  |  |  |  |
| 10 | Oily and fatty foods (any type of oil and fat such as palm, butter, ghee, olive) |  |  |  |  |  |  |
| 11 | Sugar / honey / sweet foods (any kind of sweet, cake, cake, candy, sweet juice, soft drink) |  |  |  |  |  |  |
| 12 | Pickles / Chutneys / Spices (Chili, Cap, Mustard, Soy) |  |  |  |  |  |  |

**Questionnaire on maternal health knowledge**

|  | Respondent will be able to explain correctly (0 = don't know / didn't answer / gave wrong answer; 1 = very little correct (<25% correct answer), 2 = less correct (<50% correct answer), 3 = fairly correct (50% correct answer), 4 = well correct (> 50% correct answer), 5 = well correct (> 75% correct answer) |  |
| --- | --- | --- |
| 1 | What are the symptoms of underweight / malnutrition?  Weakness / Weakness: Unable to do normal work, study, sports (disability); Weakness in disease prevention management (easily falls ill / serious illness); Weight loss / thinning; Children are not developing as much as they should; The baby's height or growth was not enough |  |
| 2 | Do children of 8-13 years take any kind of nutritional tablets / supplements?  Iron follicle supplementation; Vitamin A tablets; Worm tablets; Iodine salt |  |
| 3 | What are the common symptoms of iron deficiency? / What are the symptoms of anemia?  Lack of strength/weakness; Also in Anta / Panduvarna; Thin/ numb fingers; Frequent illness (low immunity to infection) |  |
| 4 | What are the symptoms of vitamin A deficiency?  Feeling weak / weak; Frequent illness (low immunity against infection); Eye problems, night blindness, dry eyes, corneal damage, blindness |  |
| 5 | What kind of salt should you use?  Iodized; Not iodized |  |
| 6 | When is the emergency time to wash your hands?  Before making food; Before baby meals; Before the meal itself; After the bathroom; After the child's defecation; Dirt- After moving the garbage |  |
| 7 | How do you usually purify water for safe drinking?  Boil; Chlorination; Clean with cloth; Filtering Disinfect by sunlight; Sit down |  |
| 8 | What are the steps to be taken for collection and transportation of drinking water?  Safe water source; Wash pots thoroughly before collecting water; Cover the pot; Keep water high |  |
| 9 | What are the things to keep in mind before preparing and storing food?  Wash hands with soap; Rice / vegetables are washed with safe water; Food and water are to be covered with lids; Keep food on high |  |
| 10 | How to prevent germs in food?  Use hygienic latrines, wash hands with soap (after bowel movements and after child defecation); Throw away baby's stool; Covering food and water; |  |
| 11 | How to use and maintain the toilet?  Hygienic toilet; Keep soap / sandals near the toilet; Clean pan; Good condition of sub / infrastructure; Ring-rings should not leak or be covered with soil |  |
| 12 | What kind of food should be taken to keep the body healthy?  Starch; Non-vegetarian; Fat; Vegetables and fruits; Vitamins and mineral salts; Water |  |
| 13 | Seasonal health of girl child / student  Using baby napkins / dry cloth pads during menstruation; Does the child take extra cleaning during the season (cutting unwanted hair, using clean clothes, washing well); During the season, give the child any extra food separately |  |

Interviewer Name: Signature:

Interviewee name: Signature:

Supervisor Name: Signature:

**cÖkœgvjv**

**cwimsL¨vb wefvM, ivRkvnx** **wek¦we`¨vjq**

(we:`ª: Avcbvi †`Iqv Z_¨ m¤ú~Y© †Mvcb ivLv n‡e Ges ïaygvÎ M‡elYvi Kv‡R e¨envi Kiv n‡e)

**evsjv‡`‡ki ivRkvnx ‡Rjvi c«vßeq¯‹‡`i g‡a¨ cywói Ae¯’v m¤ú‡K© Rwic: GKwU M…n¯’vwj M‡elYv**

**WvUv Gw›Uª µwgK bs:**

DËi`vZvi bvgt

wVKvbv: Dc‡Rjvt BDwbqbt IqvW©t MÖvgt

| cwiev‡ii †gvevBj bs |  |
| --- | --- |
| mvÿv‡Zi ZvwiL |  |

**mvaviY Z_¨vw`t**

| ¯^vgxi bvg |  | eqm | ‡ckv | wkÿv |
| --- | --- | --- | --- | --- |
|  |  |  |
| ¯¿xi bvg |  | eqm | ‡ckv | wkÿv |
|  |  |  |

**cwiev‡ii m`m¨‡`i A¨vb_ªc‡gwU· Ae¯’v**

| µ:b: |  | **Rb¥ ZvwiL** | **eqm** | **wj½** | **IRb** (†KwR) | **D”PZv** (†mw›UwgUvi) | **MUAC** |
| --- | --- | --- | --- | --- | --- | --- | --- |
| 1 | ¯^vgx: |  |  |  |  |  |  |
| 2 | ¯¿x |  |  |  |  |  |  |
| 3 | mšÍvb-1 |  |  |  |  |  |  |
| 4 | mšÍvb-2 |  |  |  |  |  |  |
| 5 | mšÍvb-3 |  |  |  |  |  |  |
| 6 | mšÍvb-4 |  |  |  |  |  |  |
|  |  |  |  |  |  |  |  |
|  |  |  |  |  |  |  |  |

**eqm:** (c~Y© eQi, BwcAvB I Rb¥wbeÜb KvW© †`Lv, GbAvBwW)

**cwiev‡ii m`m¨ msLv**

| **0- 5 eQi** | | **6-14 eQi** | | **15-49 eQi** | | **50-64 eQi** | | **65+eQi** | | **‡gvU** | |
| --- | --- | --- | --- | --- | --- | --- | --- | --- | --- | --- | --- |
| ‡Q‡j | ‡g‡q | ‡Q‡j | ‡g‡q | cyiæl | bvix | cyiæl | bvix | cyiæl | bvix | cyiæl | bvix |
|  |  |  |  |  |  |  |  |  |  |  |  |

**cwiev‡ii Av_©-mvgvwRK Z_¨vw`**

| 1 | cwieviwU wK Avw`evmx Rb‡Mvôxi AšÍ©f~³? 1= n¨vu, 2= bv |  |
| --- | --- | --- |
| 2 | ag©: 1=gymwjg, 2=wn›`y, 3=Lªx÷vb, 4 †eŠ×, 5= Ab¨vb¨ |  |
| 3 | cwiev‡i wbR¯^ Rwgi cwigvY (kZvsk)? **emZevwo: K…wl:** |  |
| 4 | evwo‡Z wK ‡Kvb ‰e`¨ywZK ms‡hvM i‡q‡Q? 1= n¨vu, 2= bv |  |
| 5 | cwieviwUi wK wbR¯^ †Kvb wUwf i‡q‡Q? 1= n¨vu, 2= bv |  |

**¯^v¯’¨vf¨vm m¤úwK©Z Z_vw`**

| 1 | mvaviYZ cwiev‡ii Lvevi cvwbi cÖavb Drm †KvbwU? 1=bjK~c, 2=K~qv, 3= cvBc jvBb, 4= cyKzi, 5= b`x, 6=Lvj, 7=wej, 8= Ab¨vb¨ (wbw`©ó) ...... |  |
| --- | --- | --- |
| 2 | DËi bjK~c/K~qv/cvBc jvBb n‡j Bnv wK wbivc` (†Mvov cvKv, fvj †Wª‡bR e¨e¯’v, Av‡m©wbK I e¨vK‡Uwiqv gy³)? 1= n¨vu, 2= bv |  |
| 3 | cwiev‡ii m`m¨iv mvaviYZ ‡Kvb ai‡Yi cvqLvbvi e¨envi K‡i _v‡Kb? 1= ¯^v¯’¨m¤§Z cvqLvbv (†mcwUK U¨v¼mn cvqLvbv/ Ad‡mU wcU cvqLvbv/ wis-¯j¨ve WvB‡i± wcU (IqvUvi wmj mn), **2=** A-¯^v¯’¨m¤§Z cvqLvbv (wis ¯j¨ve WvB‡i± wcU (IqvUvi wmj Qvov)/ SzjšZ/ MZ© cvqLvbv), 3= Db¥y³ ¯’v‡b (†Lvjv ¯’v‡b/‡Svc-Svo/b`x/Lvj/cyKzi cvo) |  |
| 4 | cwiev‡ii me m`m¨iv mKj RwUj mg‡q (Lvevi ˆZwii c~‡e©, LvIqvi c~‡e©, wkky‡K LvIqv‡bvi c~‡e©, cvqLvbvi c‡i, wkïi †kŠPKv‡h©i ci, AveR©bv aivi ci) mvevb w`‡q nvZ ay‡Z _v‡K? 1= mKj mgq, 2= gv‡S gv‡S, 3= KLbI bq |  |
| 5 | nvZ‡avqvi ¯’v‡b cvwb I mvevb i‡q‡Q (Z_¨ msMÖnKvixi ch©‡eÿY †_‡K)? 1= n¨vu, 2= bv |  |

**cwiev‡ii Avq I e¨‡qi wPÎ (gvwmK)**

| µ: | **Avq** | **UvKv** | µ: | **e¨q** | **UvKv** |
| --- | --- | --- | --- | --- | --- |
| 1 | ‡eZb fvZvw` |  | 1 | evmv fvov |  |
| 2 | K„wl (avb, Mg, cvU RvZxq) |  | 2 | Lv`¨-Pvj/evRvi |  |
| 3 | mwâ |  | 3 | wkÿv |  |
| 4 | QvMj/ Miæ/ †fov |  | 4 | ¯^v¯’¨/Jla/ wPwKrmv LiP |  |
| 5 | ‡gviM/KeyZi/nvm |  | 5 | cwienb |  |
| 6 | nv‡Zi KvR |  | 6 | ‡gvevBj wej |  |
| 7 | cÖevmx ‡iwgU¨vÝ |  | 7 | we`¨yZ/M¨vm/KvV/R¡vjvbx |  |
| 8 | e¨emvq |  | 8 | Avmeve cÎ/ |  |
| 9 | Aby`vb |  | 9 | C`/ Drme |  |
| 10 | FY |  | 10 | FY cwi‡kva |  |
| 11 | Ab¨vb¨ |  | 11 | mÂq |  |
|  |  |  | 12 | U¨v· |  |
|  |  |  | 13 | Dcnvi |  |
|  |  |  | 14 | Ab¨vb¨ |  |
|  | ‡gvU UvKv |  |  | ‡gvU UvKv |  |

**cwiev‡ii Lv`¨ wbivcËv**

| 1 | MZ 30 w`‡bi g‡a¨ wK Lv`¨ ‡Kbvi UvKv bv _vKvi Rb¨ Lv‡`¨i Afv‡e cwiev‡i ‡Kn †Kvb Lvevi MÖnY K‡iwb? 1= n¨vu, 2= bv |  |
| --- | --- | --- |
| 2 | MZ 30 w`‡bi g‡a¨ KZevi G NUbv N‡U‡Q? 1= K`vwPr (1-2 evi), 2= KLbI KLbI (3-10 evi), 3= cÖvqkB (10 Gi AwaK evi) |  |
| 3 | MZ 30 w`‡bi g‡a¨ wK ch©vß Lv`¨ bv _vKvq Avcwb ev cwiev‡i ‡Kn †Kvb Lvevi MÖnY QvovB ÿzavZ© Ae¯’vq ivwÎ‡Z Nygv‡Z wM‡q‡Q? 1= n¨vu, 2= bv |  |
| 4 | MZ 30 w`‡bi g‡a¨ KZevi G NUbv N‡U‡Q? 1= K`vwPr (1-2 evi), 2= KLbI KLbI (3-10 evi), 3= cÖvqkB (10 Gi AwaK evi) |  |
| 5 | MZ 30 w`‡bi g‡a¨ wK ch©vß Lv`¨ bv _vKvq Avcwb ev cwiev‡i ‡Kn †Kvb Lvevi MÖnY QvovB ÿzavZ© Ae¯’vq w`b-ivwÎ‡Z Nygv‡Z wM‡q‡Q? 1= n¨vu, 2= bv |  |
| 6 | MZ 30 w`‡bi g‡a¨ KZevi G NUbv N‡U‡Q? 1= K`vwPr (1-2 evi), 2= KLbI KLbI (3-10 evi), 3= cÖvqkB (10 Gi AwaK evi) |  |

**cwiev‡ii Lv`¨ Dcv`vbmg~n (MZ 24 N›Uvq wK wK ai‡Yi Lvevi †L‡q‡Q)**

| **#** | **Lv`¨ Dcv`vbmg~n**  (cÖ‡hvR¨ ¯’v‡b wUK wPü w`b) | mKv‡ji bv¯Ív | ¯œ¨vKm | `ycy‡ii Lvevi | ¯œ¨vKm | iv‡Zi Lvevi | ¯œ¨vKm |
| --- | --- | --- | --- | --- | --- | --- | --- |
| 1 | ‡k¦Zmvi/ cÖavb Lvevi (fvZ, Mg, iæwU, RvD, f~Uªv) |  |  |  |  |  |  |
| 2 | g~j Ges K›` (Avjy, wgwó Avjy, Avjyi Wvj) |  |  |  |  |  |  |
| 3 | kvK-mwâ (MvRi, IKiv, jvD, Kzgov, gvkiæg, g~jv, U‡gv‡Uv, evav/dzj Kwc, mxg) |  |  |  |  |  |  |
| 4 | djg~j (Kjv, †cqviv, Avg, Avbvim, Rvg, ZigyR, Kvgiv½v, Av‡cj, AvZvdj, KvVvj) |  |  |  |  |  |  |
| 5 | gvsk (Miæ, QvMj, gyiMx) |  |  |  |  |  |  |
| 6 | wWg (gyiMx, nvm, †Kv‡qj) |  |  |  |  |  |  |
| 7 | gvQ Ges mvgyw`ªK Lv`¨ (†h †Kvb ai‡Yi mvgyw`ªK gvQ, ïUKx, KvKov) |  |  |  |  |  |  |
| 8 | Wvj/ ev`vg RvZxq Lvevi (†hgb ev`vg, mqvweb, gïi, gyM, gvmKvjvB) |  |  |  |  |  |  |
| 9 | `ya Ges `y»RvZ Lv`¨ (†h †Kvb ai‡Yi `ya Ges `y»RvZ Lv`¨ †hgb `B, AvBmwµg) |  |  |  |  |  |  |
| 10 | ‰Zj Ges Pwe© RvZxq Lvevi (†h †Kvb ai‡Yi ‰Zj Ges Pwe© †hgb cvg, bbx, wN, RjcvB) |  |  |  |  |  |  |
| 11 | wPwb/ gay/ wgwó RvZxq Lvevi (†h †Kvb ai‡Yi wgwó, †KK, wcVv, K¨vwÛ, wgwó RvZxq Rym, kieZ) |  |  |  |  |  |  |
| 12 | AvPvi/ PvUbx/ gmjv RvZxq Lvevi (wPwjm, †KP¨vc, mwilv, mqvmm) |  |  |  |  |  |  |

**gv‡qi ¯^v¯’¨MZ Ávb welqK cÖkœgvjv**

|  | **DËi`vZv mwVKfv‡e e¨vL¨v Ki‡Z mÿg n‡eb** (0= Rvwb bv/ DËi †`q bvB/ f~j DËi w`‡q‡Q; 1= Lye Kg mwVK (<25% mwVK DËi), 2= Kg mwVK (<50% mwVK DËi), 3= †gvUvgywU mwVK (50% mwVK DËi), 4= fvjfv‡e mwVK (>50% mwVK DËi), 5= fvjfv‡e mwVK (>75% mwVK DËi) |  |
| --- | --- | --- |
| 1 | **Aí IR‡bi/ Acywói jÿYmg~n wK wK?**  kw³i Afve/ `ye©jZv: ¯^vfvweK KvR, covïbv, †Ljva~jv Ki‡Z cv‡i bv (AÿgZv); ‡ivM cÖwZ‡iva e¨e¯’vcbvq `ye©jZv (mn‡RB Amy¯’ n‡q hvq/ ¸iæZ¡i Amy¯’Zv); IRb K‡g hvIqv/ wPKb nIqv; wkï‡`i hZUzKz weKvk nIqv DwPZ ZZUzKz weKvk n‡”Q bv; wkïi D”PZv ev †MÖv_ chv©ß nqwb |  |
| 2 | **6-13 eQ‡ii wkïiv wK †Kvb ai‡Yi ai‡Yi cywó m¤ú©wKZ U¨ve‡jU/mvwà‡g›U‡Ukb MÖnY K‡i _v‡Kb?**  AvqiY dwjK mvwà‡g›U‡Ukb; wfUvwgb G U¨ve‡jU; K…wgi U¨ve‡jU; Av‡qvwWb jeb |  |
| 3 | **‡jvnvi-NvUwZ RwbZ mvaviY jÿY mg~n wK wK?/ A¨v‡bvwgqvi jÿYmg~n wK wK?**  kw³i Afve/ `ye©jZv; ¤øvbZv/ cvÛze‡Y©I; wPKb/ b¨yR Av½yj (koilonychia); Nb Nb Amy¯’ nIqv (msµg‡b Kg BDwgwbwU) |  |
| 4 | **wfUvwgb G Gi Afv‡ei jÿYmg~n wK wK?**  `ye©jZv/ Kg kw³ Abyfe Kiv; Nb Nb Amy¯’ nIqv (msµg‡Yi weiæ‡× Kg BwgDwbwU); ‡Pv‡Li mgm¨v, ivZKvbv, ï¯‹ †PvL, KwY©qv bó nIqv, AÜZ¡ |  |
| 5 | **Avcwb wK ai‡Yi jeb e¨envi Kiv DwPZ?**  Av‡qvWvBRW; AvqWvBRW bq |  |
| 6 | **nvZ †avqvi Riæix mgq KLb KLb?**  Lvevi ˆZwii c~‡e©; wkïi Lvev‡ii c~‡e©; wb‡R Lvev‡ii c~‡e©; cvqLvbvi ci; wkïi †kŠPKv‡h©i ci; gqjv- AveR©bv bvov‡bvi ci |  |
| 7 | **Avcwb mvaviYZ wbivc` cvwb cvb Kivi Rb¨ cvwb †K wKfv‡e cwi‡kvwaZ K‡ib?**  dzwU‡q; †K¬vwi‡bkb; Kvco w`‡q cwi¯‹vi; wdëvwis; m~‡h© Av‡jvi Øviv RxevYbybvk Kiv; w_Zv‡bv |  |
| 8 | **Lvevi cvwb msMÖn I cwien‡bi †Kvb †Kvb wel‡q c`‡ÿc wb‡Z nq?**  wbivc` cvwbi Drm; cvwb msMÖ‡ni c~‡e© cvÎ fvjfv‡e †avqv; cvÎ †X‡K ivLv; DPzu¯’v‡b cvwb ivLv |  |
| 9 | **Lvevi ‰Zwi I msi**ÿ**‡Yi c~‡e© †Kvb †Kvb wel‡q ‡Lqvj ivL‡Z nq?**  mvevb w`‡q nvZ †avqv; wbivc` cvwb w`‡q Pvj/ mwâ.. ay‡Z nq; Lvevi I cvwb XvKbv w`‡q †X‡K ivL‡Z nq; DPzu¯’v‡b Lvevi ivLv |  |
| 10 | **Lvev‡i Rxevby cÖwZ‡iva Kiv hvq wKfv‡e?**  ¯^v¯’¨-m¤§Z cvqLvbv e¨envi Kiv, mvevb w`‡q nvZ †avqv (cvqLvbvi ci I wkïi †kŠPKv‡h©i ci); wkïi gj `~‡i †djv; Lvevi I cvwb ‡X‡K ivLv; |  |
| 11 | **wKfv‡e cvqLvbv e¨envi I i**ÿ**Yv‡e**ÿ**Y Ki‡Z nq?**  ¯^v¯’¨m¤§Z cvqLvbv; mvevb/m¨v‡Ûj cvqLvbvi wbKU ivLv; cwi¯‹vi c¨vb; Ae/DcwiKvVv‡gvi fvj Ae¯’v; wis-¯øve wj‡KR bv Kiv ev gvwU w`‡q †X‡K ivLv |  |
| 12 | **kixi wVK ivL‡Z wK wK ai‡Yi Lv`¨ MÖnY Kiv DwPZ?**  ‡k¦Zmvi; Avwgl; Pwe©; mwâ I djg~j; wfUvwgb I LwbR jeY; cvwb |  |
| 13 | **‡g‡q wkï/QvÎxi FZzKvjxb ¯^v¯’¨”P©v**  gvwmK PjvKv‡j wkïwUi b¨vcwKb/ïK‡bv Kvc‡oi c¨vW e¨envi Kiv; FZzKvjxb mg‡q wkïwU wK AwZwi³ cwi¯‹vi cwi”QbZœZvi e¨e¯’v †bb (AevwÂZ †jvg KvUv, cwi¯‹vi Kvco e¨envi, fvjfv‡e †aŠZ Kiv); FZzKvjxb mg‡q wkïwU‡K Avjv`vfv‡e AwZwi³ †Kvb Lvevi †`qv |  |

Z_¨ cÖ`vbKvixi bvg: ¯^vÿi:

Z_¨ MÖnbKvixi bvg: ¯^vÿi:

ZZ¡veavq‡Ki bvg: ¯^vÿi:
